# A Perspective on a Possible New Role for Bevacizumab Combined with Paclitaxel in Hormone-Resistant Metastatic Breast-Cancer Patients

**DOI:** 10.3390/jcm15166172

**Published:** 2026-08-09

**Authors:** Giacomo Allegrini, Giulia Acconci, Gianna Musettini, Luigi Coltelli, Chiara Finale, Samanta Cupini, Linda Bartalini, Paola Orlandi, Guido Bocci

**Affiliations:** 1 Department of Oncology, Azienda USL Toscana Nord Ovest, 56121 Pisa, Italy; giulia.acconci@uslnordovest.toscana.it (G.A.); gianna.musettini@uslnordovest.toscana.it (G.M.); luigi.coltelli@uslnordovest.toscana.it (L.C.); chiarafinale@uslnordovest.toscana.it (C.F.); samanta.cupini@uslnordovest.toscana.it (S.C.); linda.bartalini@uslnordovest.toscana.it (L.B.); 2 Division of Medical Oncology, Livorno Hospital, Azienda USL Toscana Nord Ovest, 57124 Livorno, Italy; 3 Division of Medical Oncology, Pontedera Hospital, Azienda USL Toscana Nord Ovest, 56025 Pontedera, Italy; 4 Department of Clinical and Experimental Medicine, University of Pisa, 56126 Pisa, Italy; paola.orlandi@unipi.it; 5 Department of Translational Research and New Technologies in Medicine and Surgery, University of Pisa, 56126 Pisa, Italy

**Keywords:** bevacizumab, paclitaxel, pharmacogenetic, breast cancer, VEGF

## Abstract

Cyclin-Dependent Kinase 4/6 inhibitors (CDK 4/6i) represented a paradigm shift in the treatment of patients with metastatic hormone-receptor-positive (HR+), HER2-negative (HER2-) breast cancer patients. When disease progression occurs and further hormonal manipulations or target therapies fail, chemotherapy remains a therapeutic choice. For these reasons, resources should be invested in redesigning the best treatment strategy with the current chemotherapy drugs available. Nevertheless, the preferred strategy typically remains a planned sequence of single chemotherapeutic agents. This approach endeavors to extend overall survival and maintain quality of life. Within this complex and evolving therapeutic scenario, where the necessity for effective and treatment options is crucial, we explore in the present paper a perspective for a hypothetical new role of bevacizumab combined with paclitaxel for patients with metastatic HR+, HER2- breast cancer patients resistant to hormone therapy. Recent findings from our preliminary published pharmacogenetic studies, could suggest the efficacy of bevacizumab as linked to a well-defined genomic profile of genes implicated in the process of neoangiogenesis (e.g., favorable profile: *VEGF-A* rs833061/*VEGFR-2* rs1870377: CT/AT, CT/AA, TT/AA, TT/TT, CC/TT), concluding the present perspective underlying the fact that bevacizumab combined with paclitaxel will be reconsidered with this new potential use only if well-designed prospective trials confirm what has been observed from our retrospective data.

## 1. Introduction

Every year worldwide, over 110,000–130,000 new cases of HR+ advanced breast cancer occur [1]. For the HER2- breast cancer patients, the first treatment approach includes a treatment with the CDK 4/6i palbociclib, ribociclib or abemaciclib [2,3,4,5]. However, despite these advanced therapeutic options, a significant challenge arises when the disease inevitably develops resistance to CDK 4/6i, followed by further hormonal manipulations or new agents. When this occurs, chemotherapy becomes a treatment opportunity. The choice generally falls on a planned sequence of single chemotherapeutic agents. This sequential administration strategy is designed to maximize the duration of disease control, minimizing cumulative toxicity. Thus, when the disease progresses during the current chemotherapy treatment, the next single agent is introduced. This strategy aims to prolong overall survival and preserve quality of life for as long as possible. It is within this challenging and evolving therapeutic landscape, where the need for effective and durable treatment options is desirable, that we have tried to revisit the role of bevacizumab combined with paclitaxel, as a viable treatment for patients with advanced HR+, HER2- breast cancer, in light of observations from our recently published pharmacogenetic analyses. Previously, we aimed to determine whether bevacizumab combined with paclitaxel therapy could provide a tailored approach to patients, guided by their unique genetic profile, potentially minimizing the toxicities as well as achieving enhanced treatment efficacy for this setting of patients who experienced disease progression on hormone therapies, when a shift towards chemotherapy is deemed clinically unavoidable. In this paper, starting with a brief look at the current standard of treatment for metastatic hormone-positive cancer patients, which is not the main purpose of this work, we describe the steps that led us to investigate if bevacizumab combined with paclitaxel could be reconsidered due to hypothetically distinct genetic profiles, predictive of response, emphasizing the limits of our observations based on retrospective data and concluding that only a well-designed prospective study could confirm our findings. So, our aim for this perspective work is to report final considerations and the limits of approach we adopted and to consider the possible next future research developments. For detailed information on the patients’ clinical and pathological characteristics and on the specific analyses performed, we suggest consulting our three previous publications.

## 2. The New Era of the Treatment of HR+/HER2- Advanced Breast Cancer: The Central Role of CDK 4/6i

In contemporary clinical practice, first-line combinations of CDK 4/6 inhibitors with aromatase inhibitors have demonstrated profound clinical benefits, yielding an encouraging median conditional probability of survival reaching approximately 50 months [6] (Table 1). Despite these unprecedented outcomes [2,3,4,5,7,8,9], a significant management complexity arises from the eventual development of resistance, with a median time of progression-free-survival typically lasting around two years. The introduction of the CDK 4/6i in the adjuvant setting [10] has yielded an additional beneficial effect. While CDK 4/6i have demonstrated significant clinical benefits, including an encouraging median conditional probability of survival reaching approximately 50 months [6] in particular if combined with aromatase inhibitors [2,3,4,5,7,8,9] (Table 1), it is crucial to acknowledge the limitations and complexities associated with their use and the subsequent management of patients. A significant challenge remains the duration of response, with a median time of progression-free-survival typically lasting around two years. The introduction of the CDK 4/6i in the adjuvant setting has yielded an additional beneficial effect in decreasing the likelihood of relapse, but, when distant relapse occurs, we are faced with a new therapeutic challenge. So, the development of resistance during adjuvant [10] or a first-line therapies has highlighted the further need for effective strategies to manage disease progression after CDK 4/6i.

## 3. Beyond Cdk 4/6i: New Targeted Therapies

The introduction of Phosphoinositide 3-kinase-Akt-mammalian target of rapamycin (PI3K-Akt-mTOR [PIK3CA]) inhibitors and Selective Estrogen Receptor Degraders (SERDs) post CDK 4/6i and Poly (ADP-ribose) polymerase (PARP) inhibitors represents a further improvement in the treatment of HR+ metastatic breast cancer; however, their use is restricted to a subset of patients with driver mutations for these drugs. In Table 2, the results of comparative studies are briefly summarized [11,12,13,14,15,16,17,18]. Although these treatments show more efficacy when compared with controls, particularly in terms of median progression-free survival (mPFS), the limitations prevent their widespread use. Firstly, the incidence of driver mutations is not so high as would be desirable, reducing the population of patients eligible for these treatments. Subsequently, the duration of response raises concern. As result, most of the patients experiencing disease progression during a CDK 4/6i are unable to obtain a durable benefit from these drugs.

## 4. When Hormone-Resistant Disease Occurs: The Role of Chemotherapy

When disease becomes hormone resistant or not more susceptible to other treatments, an extremely widespread vision is rooted in the concept that patients’ survival is simply due to whether or not they received any form of chemotherapy at all during their subsequent treatment course. While the rise of personalized therapies tailored to individual genomic profiles and tumor characteristics can offer undeniable advancement in cancer therapy, this shift in focus may have prematurely overshadowed the continuing, and indeed crucial, role of chemotherapy in the overall treatment paradigm. The paucity of recent advancements in chemotherapy-based treatments for advanced breast cancer is, in general, a matter of concern. Considering the extended period since the last time the United States Food and Drug Administration (FDA) approved a chemotherapeutic agent for advanced breast cancer, eribulin in 2010 [19,20], a troubling pattern is emerging: a gradual, yet undeniable, decline in interest and investment in the development of novel chemotherapeutic drugs. In this context, we must not lose sight of the fact that the survival benefit associated with these initial CDK 4/6i based-regimens is invariably linked to a strategy of sequential therapies employed upon disease progression. This sequence almost universally includes the strategic and judicious use of various treatments, and, quite critically, chemotherapy remains the first option. The introduction of novel antibody-drug conjugate (ADC) treatments represents a cornerstone in the therapeutic landscape of HR+/HER2- hormone-resistant breast cancer patients and, if ongoing studies in the first-line setting confirm their efficacy over chemotherapy, a new treatment will be available [21].

However, at present, only trastuzumab deruxtecan and sacituzumab govitecan received endorsement by the FDA and the European Medicines Agency (EMA) after first-line chemotherapy [22,23]. In this rapidly evolving landscape, the remarkable activity of ADCs in the HR+ population undoubtedly redefines post-CDK4/6i sequencing [24,25]. Nevertheless, the repositioning of a pharmacogenomically-guided bevacizumab-plus-paclitaxel regimen remains highly relevant for several critical reasons. First, ADCs are associated with distinct, sometimes severe, toxicities (such as interstitial lung disease or severe neutropenia) and high financial costs, which may limit their immediate or universal accessibility in daily clinical practice globally. Second, evaluating a patient’s specific angiogenic genomic profile via MDR allows for a highly personalized, non-cross-resistant chemotherapeutic option before or between ADC lines. Rather than competing with ADCs, a pharmacogenomically optimized bevacizumab–paclitaxel regimen expands the therapeutic armamentarium, offering a highly effective, tailored, and economically sustainable ‘potential new role’ for a molecularly selected subgroup of patients who have failed endocrine manipulations.

Therefore, in this context, premature dismission or minimization of the importance of chemotherapy is a risky assumption. Chemotherapy remains a vital tool in the armamentarium for managing advanced breast cancer patients.

While the specific clinical presentation of each patient rightly guides the clinician in making the most appropriate treatment decision, it is equally evident that adoption of mono-chemotherapy is emerging with a combination chemotherapy, proposed only for patients with high tumor burden or visceral crisis [26].

Although current clinical practice favors sequential mono-chemotherapy for most patients, reserving combinations exclusively for visceral crises [26], this rigid paradigm often restricts personalized treatment depth. Consequently, re-evaluating synergistic, biology-driven combinations becomes essential to bridge the gap between patient expectations and durable disease control [27]. In fact, clinical evidence in general fails to demonstrate an advantage in terms of overall survival compared to single-drug chemotherapy.

The limit of this approach is the risk of discontinuing any form of research aimed at improving chemotherapy-based treatments. Furthermore, treating patients with a carefully selected combination of multiple chemotherapy drugs often yields significantly better results in terms of response rate and PFS than relying on a single drug alone, a fact that is often overlooked when focusing solely on the mono-chemotherapy [27]. Indeed, this focus on simply using any chemotherapy disregards the potential for synergistic effects and the ability to target multiple pathways of cancer cell growth simultaneously that are possible with combination therapies.

## 5. Bevacizumab in Advanced HR+/HER2- Breast Cancer: Has It All Already Been Written?

The clinical use of bevacizumab for the treatment for metastatic breast cancer, specifically from its first approval to subsequent withdrawal, represents a cautionary tale in oncology drug development and regulation. The initial results from clinical trials including bevacizumab, a monoclonal antibody targeting vascular endothelial growth factor-A (VEGF-A) and blocking signaling through vascular endothelial growth factor receptor 1 (VEGFR-1) and VEGFR-2 on endothelial cells of tumor microenvironment [28], seemed so significant that in 2008, the FDA granted accelerated approval for its use in combination with paclitaxel for patients with metastatic breast cancer [29].

This decision was largely based on results from the phase III study E2100 trial, which demonstrated a significant improvement in mPFS compared to paclitaxel alone [30,31].

This new combination offered an alternative option for patients with metastatic disease. The expectation was that inhibiting angiogenesis, the formation of new blood vessels that tumors need to grow and spread, would translate into a meaningful survival benefit. However, these initial results were soon tempered by the next clinical trials. While RIBBON1 and AVADO trials [32,33] confirmed the initial findings of an improved PFS with bevacizumab in combination with different chemotherapy regimens, a crucial endpoint—overall survival (OS)—consistently failed to demonstrate a statistically significant advantage. This discrepancy between PFS and OS, a phenomenon observed in other oncology settings as well, raised serious questions about the true clinical benefit of bevacizumab [30,31,32,33] (Table 3).

So, the FDA, due to a lack of survival benefit and subsequent increased treatment-related toxicities, decided to withdraw its initial approval for bevacizumab in 2011 [34].

Many argued that when long survival post-progression (SPP) on first-line treatment is expected, as in breast cancer, the absence of a clear OS benefit may not mean that the first-line therapy failed to improve OS. If treatment truly improved PFS but did not change SPP, the likelihood of also observing a statistically significant OS difference depended on how long the median SPP was and how large the PFS gain appeared. Differences between patients and the variety of treatments given after progression reduced the OS gap between the trial arms. That reduction became bigger as median SPP increased. With a short median SPP, a significant PFS benefit usually resulted in a significant OS benefit. Longer SPP intervals introduced more variability, which diluted the treatment effect and made significant OS less likely [35].

The clinical divergence between a major PFS benefit and the lack of an OS advantage led to conflicting regulatory positions, culminating in the FDA withdrawing its approval in 2011 while the EMA maintained the indication [34,36]. This historical controversy within the oncology community highlights the fact that the historical failure of bevacizumab was not a lack of efficacy, but rather a lack of patient selection. Interest subsequently waned in the HR+ setting, due to the advent of CDK 4/6 inhibitors [2,3,4,5]; however, the future clinical utility of this anti-angiogenic combination strictly hinges on transforming it from a broad-spectrum agent to a biomarker-targeted therapy [36].

Since the initial publication of the pivotal three randomized phase III trials (Table 3) evaluating bevacizumab in breast cancer, a substantial body of research has been dedicated to identifying such predictive biomarkers. Investigations have encompassed a wide array of molecular analyses, including specific single-nucleotide polymorphisms (SNPs) within genes that play a critical role in the complex process of angiogenesis [37,38].

Despite the regulatory allowance of continuing to prescribe bevacizumab in combination with paclitaxel granted by the EMA, interest in this combination has waned considerably, especially in the setting of HR+ breast cancer patients, in light of the new treatment prospects offered by CDK 4/6i [2,3,4,5] and for triple-negative (TN) breast cancer. This is due to the introduction of immune check-point (IC) and PARP inhibitors as first-line treatment when indicated [15,18]. Given this shift in clinical practice, the future role of bevacizumab in breast cancer therapy hinges on identifying predictive biomarkers that could accurately identify patients most likely to benefit from it. In essence, the key to revitalizing bevacizumab’s utility lies in transforming it from a broad-spectrum agent to a targeted therapy applicable only to a selected and responsive subgroup of patients, through the discovery and validation of robust and reliable predictive factors. In this context, analyzing global research trends highlights the fact that integrating molecular classification with emerging clinical hotspots is crucial for refining anti-angiogenic therapeutic windows [39]. Furthermore, moving beyond single-gene paradigms toward advanced multi-parametric screening allows for more precise efficacy prediction. Modern computational workflows, including machine learning-driven biomarker algorithms, have already demonstrated significant utility in predicting personalized risks and therapy outcomes within specific breast cancer cohorts [40]. Consequently, leveraging innovative modeling strategies to cross-reference anti-angiogenic responsiveness with tailored molecular profiles represents a mandatory milestone for contemporary personalized medicine.

Furthermore, researchers have explored the potential of soluble plasma factors, such as VEGF-A itself, as predictive markers [41]. However, despite the extensive research efforts and the promising theoretical underpinnings, none of these investigated factors, whether genetics or soluble proteins, have been successfully validated in prospective clinical trials. Crucially, no biomarker has consistently and reliably predicted response to bevacizumab, not only in breast cancer but also in any of the other solid tumors where this drug is commonly used.

This lack of validated biomarkers significantly limits the ability to offer a tailored personalized treatment with bevacizumab and effectively target it to the patients who are most likely to derive benefit. The consistent failure to identify predictive biomarkers underscored the limitations of past research approaches and highlights the fact that it is imperative to explore alternative models and methodologies. The field now recognizes the need to shift the research focus towards novel avenues of investigation that may provide a more comprehensive understanding of the mechanisms driving bevacizumab response and resistance. The Multifactor Dimensional Reduction (MDR) is a methodological approach conceived and developed by Moore and collaborators [42,43,44], with the purpose of identifying a specific and informative set of genetic profiles. These profiles, ideally, would serve as predictive biomarkers, enabling clinicians to forecast the likely efficacy of chemotherapy treatments as well as the capecitabine-and-oxaliplatin regimen in patients diagnosed with metastatic colorectal cancer [45]. This predictive capacity holds significant promise for personalized medicine, allowing for more tailored and effective treatment strategies. Recognizing the potential of pharmacogenomics in predicting treatment outcomes, our research team embarked on a series of comprehensive pharmacogenomics analyses. The initial step involved the careful selection of a panel of genes known to be critically implicated in the process of tumor angiogenesis [46,47,48]. The overarching objective of these analyses was to evaluate the possibility of identifying a distinct genetic profile capable of predicting the best probability of efficacy of a regimen consisting of bevacizumab in combination with paclitaxel in patients with advanced, HER2- breast cancer. The rationale driving this research approach arises from the accumulated evidence indicating the complex relationship between angiogenesis molecular players and bevacizumab response [44]. Given the complex interplay of multiple genes in many biological and pharmacological processes, particularly in the development of diseases and response to therapies, understanding gene–gene interactions is crucial. Traditional methods often analyze isolated genes, ignoring the synergistic or antagonistic effects that can arise when multiple genes act together. This limitation highlighted the need for analytical approaches that could explicitly consider these interactions. Consequently, the idea arose to leverage a method, such as MDR methodology, which is specifically designed to capture and model the intricate relationships between multiple genetic factors. MDR and similar techniques offer the potential to identify combinations of genes that, while individually having little impact, collectively exert a significant influence on a phenotype or disease outcome, a phenomenon defined as nonlinear interaction or statistical epistasis [49]. By moving beyond single-gene analysis, these methods promise a more comprehensive and nuanced understanding of the genetic architecture underlying complex traits.

## 6. MDR Methodology Applied to Identify Genetic Profile Predictive of Bevacizumab’s Response: A Hypothesis for a New Research Direction

In this final section, which represents the core of the present paper, we review the elements we consider most relevant from the analyses conducted and discussed in the three previously published papers. We acknowledge the limitations encountered, which are primarily related to the available data and are well described in previous articles.

The main limit is the nature of the analysis itself, a cohort of patients with metastatic breast cancer treated with bevacizumab in combination with paclitaxel, who were compared with a historical cohort treated with paclitaxel alone.

The results of our published previous analysis with the multifactor dimensionality reduction (MDR) methodology are briefly summarized in Table 4, while details of how MDR works, the analyses, and patient characteristics are reported in the original papers [50,51,52]. Briefly, a well-established validated methodology, MDR, used to identify a genetic profile and with the ability to predict the drug response, was applied to our analysis to identify the potential genetic profile able to predict the bevacizumab response. As expected, the multi-parametric MDR model identified distinct genetic profiles across the evaluated cohorts. In the initial exploratory study with 113 patients, a favorable profile involving *VEGFR-2* rs11133360 and *IL-8* rs4073 SNPs demonstrated a significant predictive value, yielding a median PFS (mPFS) of 14.1 months, compared to 10.2 months for the unfavorable group (PFS HR 0.44, 95% CI 0.29–0.66; *p* < 0.0001), which translated into a median OS (mOS) of 35.7 vs. 24.3 months (OS HR 0.61, 95% CI 0.43–0.86; *p* = 0.096). Crucially, as the research advanced toward larger and more homogenous cohorts, the definition of the favorable genotype shifted, to encompass the *VEGFA* rs833061 and *VEGFR-2* rs1870377 interaction. This evolution in biomarker selection reflects the mathematical refinement of the MDR algorithm when processing larger sample sizes, transitioning from early multi-gene exploratory flags to a more stable, core angiogenic signature. Utilizing this refined *VEGFA*/*VEGFR-2* profile in a balanced cohort of 215 patients, the favorable signature correlated with an improved mPFS of 16.8 vs. 10.6 months (HR 0.64, 95% CI 0.50–0.90; *p* = 0.007) and a strong trend toward an OS advantage of 39.6 vs. 27.9 months (HR 0.71, 95% CI 0.50–1.01; *p* = 0.058). When strictly isolating the HR+ population, this retrospective benefit became even more pronounced, showing a striking mPFS of 22.9 vs. 8.7 months (HR 0.44, 95% CI 0.28–0.69; *p* = 0.0001) and an mOS of 50.2 vs. 23.5 months (HR 0.40, 95% CI 0.25–0.66; *p* = 0.003).

Based on these results, an expanded study incorporated a numerically adequate control group comprising patients treated with paclitaxel without bevacizumab [47]. A proper number of patients in control arm allowed us to evaluate a possible assessment of the independent contribution of bevacizumab on treatment effects. And this approach allowed us to hypothesize a more accurate determination of a predictive power of the MDR-derived genetic profiles [47]. However, one of the principal limitations, extensively examined and discussed in previous published work, were conclusions based on an analysis performed on a retrospective series of patients in which one group was treated with a combination of bevacizumab and paclitaxel and the other with paclitaxel alone, using historical cohorts. A comparison of clinical and pathological characteristics did not reveal statistically significant differences between the groups, nor notable differences between patients with a favorable genetic profile and those with an unfavorable profile [50]. Although the differences seen between the groups may stem from the retrospective design of the study, the favorable and unfavorable groups had similar characteristics.

As we were aware of this limitation, the inclusion of a paclitaxel-alone control group provided a possible benchmark to measure the incremental benefit conferred by bevacizumab in the context of specific genetic backgrounds. A specific genetic profile, involving the *VEGF-A* rs833061/*VEGFR-2* rs1870377 SNPs, was identified in patients treated with bevacizumab and paclitaxel, which correlated with improved PFS which translated into a hypothetical OS advantage [51]. Building upon this major observation, the exploration of the influence of HR status on the efficacy of bevacizumab and paclitaxel, in conjunction with the pharmacogenetic profile, was the next step of the clinical research [52]. Indeed, in the HR+ population, the survival advantage associated with the favorable genetic profile seemed more pronounced than in the whole population of patients [52]. In contrast, in the triple-negative subgroup with the previously identified genetic profile, we did not observe any apparent survival benefit [52]. Moreover, the observation that the favorable pharmacogenetic profile, in terms of the *VEGF-A* rs833061/*VEGFR-2* rs1870377 SNPs, was present in over 70% of patients experiencing an mPFS exceeding 20 months, was particularly noteworthy [52]. Furthermore, the study suggested a possible and hypothetical correlation between this extended PFS and a corresponding improvement in OS, with the suggestion of achieving a median OS greater than 50 months [52]. Although intriguing, these findings raise questions about the potential for personalized treatment strategies based on genetic profiling.

## 7. Future Perspectives

The results of these recent analyses may indicate the hypothetical presence of a favorable genetic profile capable of identifying patients most likely to achieve improved survival with bevacizumab combined with paclitaxel, in a population largely characterized by disease progression after first-line hormonal therapy with aromatase inhibitors and, more generally, within the hormone-resistant population. However, these observations remain speculative because of the limitations described in the introduction and aspects of the study conduct, including its ambidirectional design, the absence of blinding, a potentially small sample size for the MDR analysis, the lack of patients previously treated with CDK4/6 inhibitors, and the arbitrary exclusion of patients with progression-free survival (PFS) between 10 and 20 months. These issues introduce biases that prevent drawing conclusions that would change clinical practice. Moreover, the small size of the control group treated with paclitaxel alone requires considerable caution in interpreting the role of genetic profiles in this patient population. Certainly, some elements may have caused more noticeable bias than others. For example, we focused on evaluating the MDR analysis in HR+ tumors by introducing time-to-progression as a new variable, selecting patients with median progression-free survival (mPFS) greater than 20 months or less than 10 months. Although the empirical decision to exclude patients with mPFS between 10 and 20 months may introduce bias, it was based on the hypothesis that the marked clinical benefit observed in a subset of patients treated with bevacizumab plus paclitaxel—who had a median survival of up to 50 months—reflected an underlying genetic profile predictive of a response to bevacizumab that the MDR methodology could identify, while acknowledging the limitations of the MDR approach.

As briefly reported in Table 4**,** upon increasing the homogeneity of the groups under analysis by hormone-receptor status and time-to-progression, reducing variability, the MDR methodology was potentially able to identify the favorable genetic profile with the highest probability of response to bevacizumab combined with paclitaxel in terms of efficacy, minimizing the burden of treatment-related toxicities.

Pharmacogenetics is most useful when a well-validated gene–drug pair changes a concrete decision such as drug choice, dose, or avoidance of serious toxicity, and its clinical use remains uneven because evidence strength, guidelines, infrastructure, and reimbursement differ by setting. Pharmacogenetics is most clinically applicable when the mechanism is simple and actionable: a variant changes drug metabolism, transport, or target response enough to justify a different drug or dose. A classic example in oncology includes DPYD–5-FU, where reduced or absent catabolism can make the drug more toxic and potentially fatal [53]. Based on these premises, our studies on bevacizumab pharmacogenetics through the MDR analysis are still far from resulting in a routine clinical application, but they open a new perspective for future clinical randomized trials that may help to clearly identify breast cancer patients responding to bevacizumab plus paclitaxel. Indeed, these observations could lead to speculation regarding the possibility that bevacizumab, in combination with paclitaxel, may offer a possible OS benefit in HR+ metastatic breast-cancer patients exhibiting the *VEGF-A* rs833061/*VEGFR-2* rs1870377 SNP-favorable pharmacogenetic profile. However, only a well-designed, prospective, randomized clinical trial will be able to investigate this possibility.

In conclusion, our observations remain speculative, but in case of positive results from a phase III study the implications for treatment algorithms could be profound, with a comprehensive reassessment of the current treatment strategy.

## Figures and Tables

**Table 1:** Phase III first-line studies including CDK 4/6i + Aromatase Inhibitors (AIs) vs. AI alone in HR+/HER2 breast cancer patients.

HR (95% CI); *p* Value	mOS (Months)	HR (95% CI); *p* Value	mPFS (Months)	Sample Size (N)	Treatment	Study Name	First Author [Reference]
0.80 (0.64–1.01); 0.06	66.8 vs. 53.7	0.53 (0.43–0.67); <0.0001	29.0 vs. 14.8	493	Abemaciclib + AI vs. AI	Monarch 3	Goetz et al. [4]
0.76 (0.63–0.93); 0.008	63.9 vs. 51.4	0.56 (0.45–0.71); <0.0001	25.3 vs. 16.0	668	Ribociclib + AI vs. AI	Monaleesa-2	Hortobagyi et al. [3,7]
0.95 (0.78–1.18); 0.34	53.9 vs. 51.2	0.58 (0.46–0.72); <0.0001	24.8 vs. 14.5	666	Palbociclib + AI vs. AI	Paloma-2	Finn et al. [2], Slamon et al. [8]
0.76 (0.61–0.96); 0.02	58.7 vs. 48.0	0.57 (0.44–0.74); <0.0001	27.5 vs. 13.8	495	Ribociclib + AI vs. AI	Monaleesa-7	Tripathy et al. [5], Lu et al. [9]

***Population Setting**: Data from major Phase III first-line trials. MONARCH 3, MONALEESA-2, and PALOMA-2-enrolled postmenopausal patients; MONALEESA-7-evaluated pre/perimenopausal patients. **Clinical Focus**: Therapeutic benefits are highly consistent across both premenopausal and postmenopausal cohorts, establishing the foundational modern first-line standard of care. **Abbreviations:** AI: aromatase inhibitor; CI: confidence interval; HR: hazard ratio; mOS: median overall survival; mPFS: median progression-free survival; N: number of patients*.

**Table 2:** Targeted Therapies in Phase III clinical trials post CDK 4/6i.

HR (95% CI) *p*-Value	mOS (Months)	HR (95% CI); *p*-Value	mPFS (Months)	Sample Size (N)	Mutation Prevalence	Treatment	Study Name	First Author [Reference]
0.59 (0.44–0.78); 0.0003	24.9 vs. 16.1	0.55 (0.39–0.77); 0.0005	3.8 vs. 1.9	228	~30–40% (ESR1)	Elacestrant vs. SoC (Fulv/AI)	EMERALD	Bidard et al. [11]
0.82 (0.57–1.18); 0.26	NR vs. 34.4	0.59 (0.48–0.72); <0.0001	10.9 vs. 5.5	426	100% (ER+)	Imlu + Abema vs. Imlu	EMBER-3	Jhaveri et al. [12]
0.67 (0.48–0.93); 0.019	34.0 vs. 27.0	0.43 (0.32–0.59); <0.001	15.0 vs. 7.3	325	~35–40% (PIK3CA)	Inavo + Palbo + Fulv vs. Pbo + Palbo + Fulv	INAVO120	Turner et al. [13]
0.74 (0.56–0.98); 0.03	NR vs. NR	0.60 (0.51–0.71); <0.001	7.2 vs. 3.6	708	~50% (AKT/PTEN/PIK3CA)	Capiva + Fulv vs. Pbo + Fulv	CAPItello-291	Turner et al. [14]
0.90 (0.66–1.23); 0.51	19.3 vs. 17.1	0.58 (0.43–0.80); <0.001	7.0 vs. 4.2	302	~5–10% (gBRCA)	Olaparib vs. TPC (Chemo)	OlympiAD	Robson et al. [15,16]
0.85 (0.67–1.07); 0.17	19.3 vs. 19.5	0.54 (0.41–0.71) <0.001	8.6 vs. 5.6	431	~5–10% (gBRCA)	Talazoparib vs. TPC (Chemo)	EMBRACA	Litton et al. [17,18]

***Trial Setting**: Landmark Phase III randomized clinical trials evaluating secondary targeted therapeutic regimens implemented after prior CDK 4/6 inhibitor failure. **Clinical Focus:** Therapeutic benefits are strictly biomarker-dependent and restricted to specific molecular subgroups harboring targetable driver alterations. **Abbreviations**: AI: Aromatase Inhibitor; CI: Confidence Interval; ESR1: Estrogen Receptor 1 gene; PI3KCA:* Phosphatidylinositol-4,5-Bisphosphate 3-Kinase Catalytic Subunit Alpha; *AKT*: Protein Kinase B; *PTEN*: Phosphatase and Tensin Homolog; *gBRCA: Germline BRCA mutation; HR: Hazard Ratio; mOS: Median Overall Survival; mPFS: Median Progression-Free Survival; NR: Not Reached; Pbo: Placebo; TPC: Treatment of Physician’s Choice*.

**Table 3:** Phase III studies combining bevacizumab + chemotherapy vs. chemotherapy alone.

HR (95% CI) *p*-Value	mOS	HR (95% CI) *p*-Value	mPFS	Sample Size (N)	Treatment	Study Name	First Author [Reference]
0.88 (0.75–1.05) 0.16	26.7 vs. 25.2	0.48 (0.38–0.61); <0.001	11.3 vs. 5.8	722	Bev + Pacli vs. Pacli	E2100	Miller et al. [30,31]
1.03 (0.81–1.31) 0.85	30.2 vs. 31.9	0.69 (0.54–0.87); 0.0001	10.1 vs. 8.1	736	Bev + Doce vs. Doce	AVADO	Miles et al. [33]
1.03 (0.84–1.26) 0.83	30.8 vs. 27.2	0.64 (0.52–0.78); <0.001	9.2 vs. 8.0	1237	Bev + Chemo vs. Chemo	RIBBON-1	Robert et al. [32]

***Trial Setting**: landmark phase III randomized clinical trials evaluating the addition of anti-VEGF-targeted therapy to standard first-line chemotherapy. **Abbreviations:** Bev: bevacizumab; Doce: docetaxel; Pacli: paclitaxel; Chemo: chemotherapy; CI: confidence interval; HR: hazard ratio; mOS: median overall survival; mPFS: median progression-free survival*.

**Table 4:** Efficacy Outcomes by MDR Genetic Profiles of patients treated with Paclitaxel + Bevacizumab.

HR (95% CI) *p*-Value	mOS (Months)	HR (95% CI); *p*-Value	mPFS (Months)	HR Status	Genetic Profile (N)	First Author, Year, Journal
0.61 (0.43–0.86); 0.096	35.7	0.44 (0.29–0.66); <0.0001	14.1	+/−	Favorable * (48)	Allegrini et al. [50]
	24.3		10.2	+/−	Unfavorable ” (48)	
0.71 (0.50–1.01); 0.058	39.6	0.64 (0.50–0.90); 0.007	16.8	+/−	Favorable ’ (66)	Coltelli et al. [51]
	27.9		10.6	+/−	Unfavorable ° (149)	
0.69 (0.45–1.05); 0.005	42.8	0.63 (0.40–0.99); 0.001	18.1	+	Favorable ’ (56)	Coltelli et al. [52]
	30.4		11.6	+	Unfavorable ° (119)	
0.40 (0.25–0.66); 0.003	50.2	0.44 (0.28–0.69); 0.0001	22.9	+	Favorable ^ (47)	
	23.5		8.7	+	Unfavorable == (59)	

**Trial Setting**: retrospective pharmacogenetic interaction analyses evaluating multi-parametric gene–gene profiles via Multifactor Dimensionality Reduction (MDR). **Clinical Focus:** therapeutic benefits of the anti-angiogenic combination are selectively restricted to the pharmacogenomically defined favorable cohort within hormone receptor-positive disease. **Abbreviations:** HR status: hormone-receptor status; mPFS: median progression-free survival; mOS: median overall survival CI: confidence interval; HR: hazard ratio; GENOTYPE LEGEND (MDR Interaction): (*) FAVORABLE: VEGFR-2 rs11133360/IL-8 rs4073: **TT/TT, CC/AA, CC/AT, CT/AA, CT/AT; (“) UNFAVORABLE:** VEGFR-2 rs11133360/IL-8 rs4073: **TT/AA, TT/AT, CC/TT, CT/TT; (‘) FAVORABLE:** VEGF-A rs833061/VEGFR-2 rs1870377: **CC/TT, CT/AT; (°) UNFAVORABLE:** VEGF-A rs833061/VEGFR-2 rs1870377: **TT/TT, TT/AT, TT/AA, CT/TT, CT/AA, CC/AT, CC/AA**; (^) **FAVORABLE:** VEGF-A rs833061/VEGFR-2 rs1870377: **CT/AT, CT/AA, TT/AA, TT/TT, CC/TT; (==) UNFAVORABLE:** VEGF-A rs833061/VEGFR-2 rs1870377:**CT/TT, TT/AT, CC/AT, CC/AA**.

## Data Availability

No new data were created or analyzed in this study. Data sharing is not applicable to this article.
